# Within and between classroom transmission patterns of seasonal influenza among primary school students in Matsumoto city, Japan

**DOI:** 10.1073/pnas.2112605118

**Published:** 2021-11-08

**Authors:** Akira Endo, Mitsuo Uchida, Naoki Hayashi, Yang Liu, Katherine E. Atkins, Adam J. Kucharski, Sebastian Funk

**Affiliations:** ^a^Department of Infectious Disease Epidemiology, London School of Hygiene & Tropical Medicine, London, WC1E 7HT, United Kingdom;; ^b^The Centre for Mathematical Modelling of Infectious Diseases, London School of Hygiene & Tropical Medicine, London, WC1E 7HT, United Kingdom;; ^c^The Alan Turing Institute, London, NW1 2DB, United Kingdom;; ^d^School of Tropical Medicine and Global Health, Nagasaki University, Nagasaki, 852-8523, Japan;; ^e^Japan Society for the Promotion of Science, Tokyo, 102-0083, Japan;; ^f^Graduate School of Medicine, Gunma University, Gunma, 371-8511, Japan;; ^g^Simulation & Mining Division, NTT DATA Mathematical Systems Inc., Tokyo, 160-0016, Japan;; ^h^Department of Mathematical and Computing Science, School of Computing, Tokyo Institute of Technology, Tokyo, 152-8552, Japan;; ^i^Centre for Global Health Research, Usher Institute, University of Edinburgh, Edinburgh, EH16 4UX, United Kingdom

**Keywords:** influenza, school, mathematical model, class size, social network

## Abstract

Schools play a central role in the transmission of many respiratory infections. Heterogeneous social contact patterns associated with the social structures of schools (i.e., classes/grades) are likely to influence the within-school transmission dynamics, but data-driven evidence on fine-scale transmission patterns between students has been limited. Using a mathematical model, we analyzed a large-scale dataset of seasonal influenza outbreaks in Matsumoto city, Japan, to infer social interactions within and between classes/grades from observed transmission patterns. While the relative contribution of within-class and within-grade transmissions to the reproduction number varied with the number of classes per grade, the overall within-school reproduction number, which determines the initial growth of cases and the risk of sustained transmission, was only minimally associated with class sizes and the number of classes per grade. This finding suggests that interventions that change the size and number of classes, e.g., splitting classes and staggered attendance, may have a limited effect on the control of school outbreaks. We also found that vaccination and mask-wearing of students were associated with reduced susceptibility (vaccination and mask-wearing) and infectiousness (mask-wearing), and hand washing was associated with increased susceptibility. Our results show how analysis of fine-grained transmission patterns between students can improve understanding of within-school disease dynamics and provide insights into the relative impact of different approaches to outbreak control.

## Significance

Empirical evidence on detailed transmission patterns of influenza among students within and between classes and grades and how they are shaped by school population structure (e.g., class and school sizes) has been limited to date. We analyzed a detailed dataset of seasonal influenza incidence in 29 primary schools in Japan and found that the reproduction number at school did not show any clear association with the size or the number of classes. Our findings suggest that the interventions that only focus on reducing the number of students in class at any moment in time (e.g., reduced class sizes and staggered attendance) may not be as effective as measures that aim to reduce within-class risk (e.g., mask-wearing and vaccines).

Influenza virus and other directly transmitted pathogens typically spread over social contact networks involving frequent conversational or physical contacts (1–4). There is evidence that schools are important social environments that can facilitate the transmission of influenza via close contact between students (5–9). Previous studies have collected contact data between students using questionnaires and wearable sensor devices and found strong assortativity of contact rates within classes and grades (10–14), which is likely relevant to the within-school transmission dynamics of respiratory infections and the effectiveness of school-based interventions. However, such insights from contact data also need to be validated with real-world outbreak data because contacts as measured in those studies may not necessarily be fully representative of the types of contacts that lead to transmission (e.g., with regards to proximity and duration). In this light, the differential transmission rates of influenza associated with classes and grades have also been estimated from empirical outbreak data in a few studies (6, 15, 16). However, those studies are limited to the analysis of only one or two schools and included a relatively small number of cases (<300). Therefore, robust findings across schools with different structures that capture the full range of heterogeneity in within-school transmission dynamics have remained a crucial knowledge gap.

Understanding how school population structures (e.g., class and school sizes) shape transmission dynamics is key to making predictions about outbreak dynamics and interventions in these settings. Modeling studies of school outbreaks often require a choice between the “density-dependent mixing” and “frequency-dependent mixing” assumptions (17). The density-dependent mixing assumes that the transmission rate between a pair of students is constant regardless of the class/school sizes, while the frequency-dependent mixing assumes an inverse proportionality between them. As a result, the reproduction number is expected to increase with class/school size with the density-dependent mixing assumption and remain stable with the frequency-dependent mixing assumption. Whether the transmission is best characterized by the density-dependent mixing, frequency-dependent mixing, or any other alternative assumption may vary between different modes of transmission and exposure settings (18–22). However, choices between the assumptions made by existing studies of school outbreaks vary widely and are not based on a clear empirical consensus (9, 23–26). These makes it challenging to interpret simulation studies evaluating school-based interventions (e.g., reduced class sizes) because the estimated effect sizes can heavily rely on the assumed mixing patterns (27–31).

To fill this knowledge gap in heterogeneous transmission dynamics at school, we applied a mathematical model of influenza virus transmission to a large-scale dataset from the 2014 to 2015 season in Matsumoto city, Japan, which included diagnosed influenza reports among 10,923 primary school students and their household members. The model accounted for within-school transmissions as well as introductions to and from households and risk from the general community, which constitute key social layers of transmission (32–34). Using this model, we estimated fine-scale heterogeneous transmission patterns among students within and between classes and grades, as well as determinants of transmission rates including school structures and precautionary measures.

## Results

**Fig. 1.**
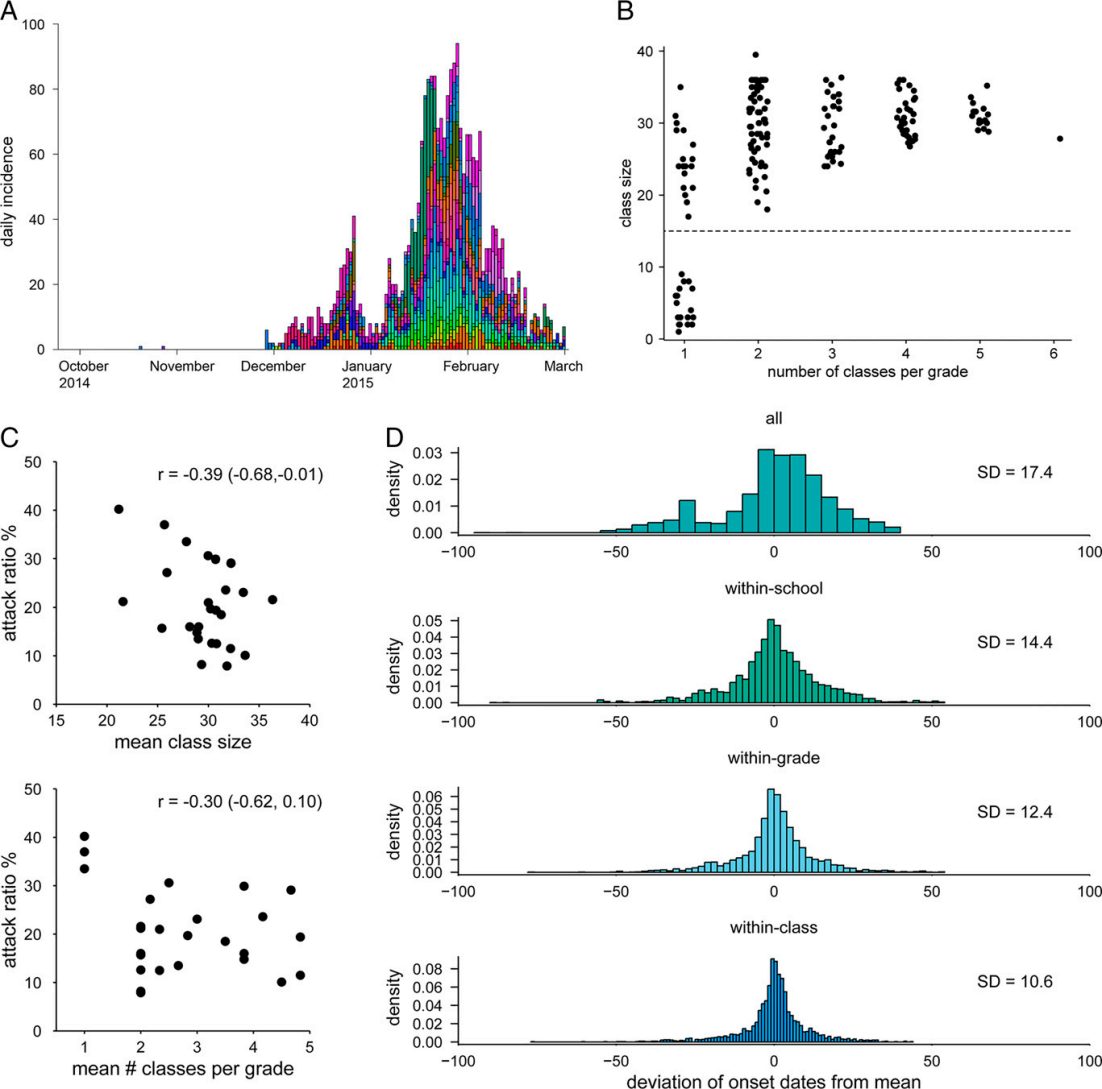
Transmission dynamics of seasonal influenza in primary schools in Matsumoto city, Japan, and estimated effects of interventions for SARS-CoV-2. (*A*) Epidemic curve of seasonal influenza by illness onset in primary schools in Matsumoto city, 2014/15. Colors represent different schools. Month names denote the first day of the month. (*B*) Scatterplot of the class sizes and the number of classes per grade in the dataset. Each dot represents a class in the dataset. Dots are jittered along the x-axis. Three schools had classes of fewer than 15 students (denoted by dotted horizontal line) and were excluded from the model fitting. (*C*) The scatterplots of the school attack ratio (%) against the mean class size and the mean number of classes per grade. The correlation indices (*r*) and 95% CIs are also shown. (*D*) Temporal clustering patterns of students’ onset dates with different levels of groupings reproduced from the school transmission model. The distributions of the deviance of each student’s onset from the group mean are displayed at overall, school, grade, and class levels. The SD of each distribution is also shown.

We analyzed citywide survey data of 10,923 primary school students (5 to 12 y old) in Matsumoto city, Japan, in 2014/15, which included 2,548 diagnosed influenza episodes among students (Fig. 1*A*). The dataset was obtained from 29 schools with a range of class structures (sizes and number of classes per grade), allowing for detailed analysis of within and between class transmission patterns (Fig. 1*B*). The attack ratio (i.e., the cumulative proportion diseased) in each school (excluding three distinctively small schools with fewer than 15 students per class) showed weak to null negative correlations with the mean class size and the mean number of classes per grade (Fig. 1*C*). The onset dates of students showed a temporal clustering pattern associated with school structure (Fig. 1*D*). When the students were partitioned into different levels of groupings (i.e., by class, grade, school and overall), the deviation of onset dates from the within-group mean tended to be smaller with finer groupings.

**Fig. 2.**
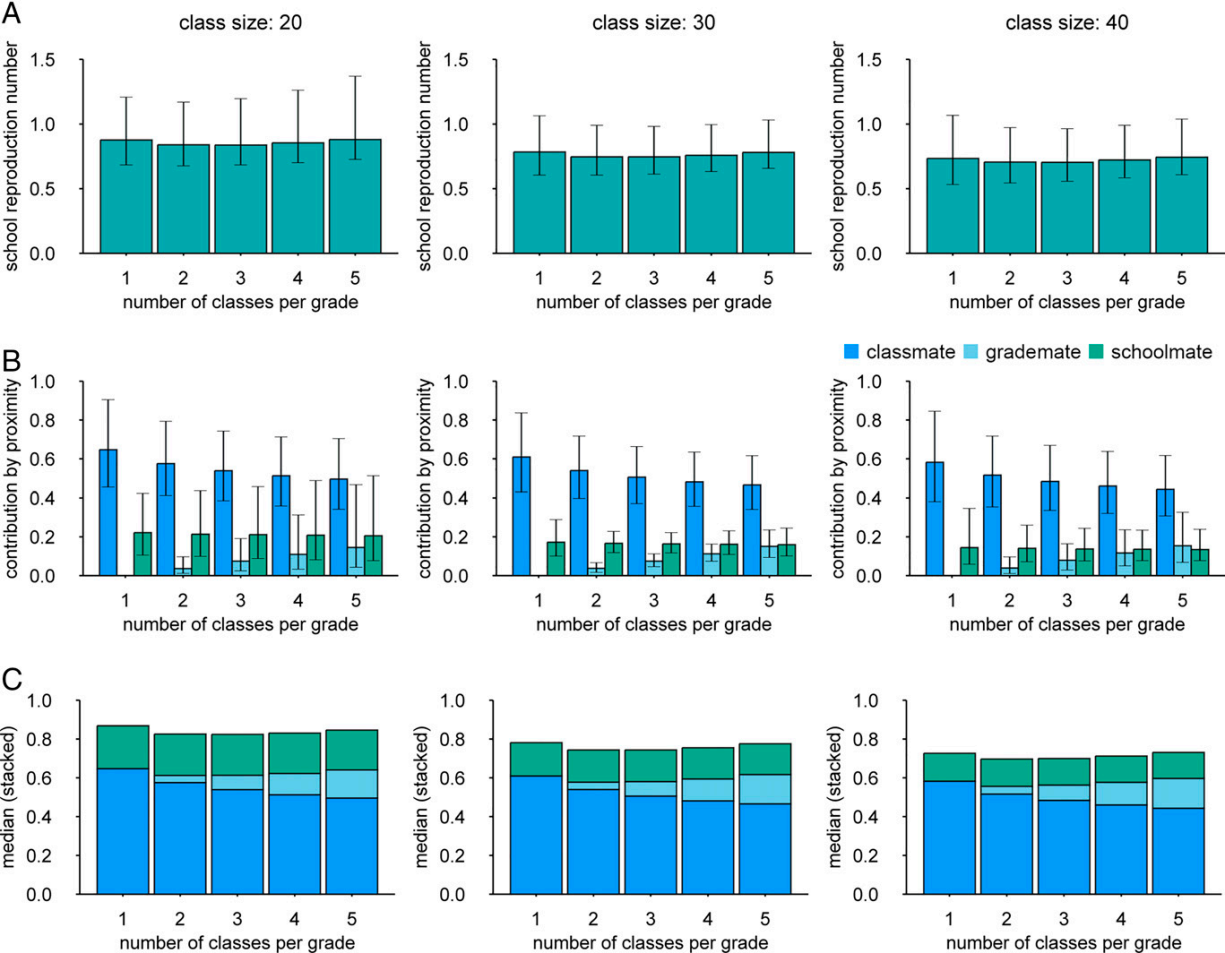
The estimated within-school transmission patterns of seasonal influenza among primary school students in Matsumoto city, Japan. (*A*) The overall school reproduction number (*R*_S_) under different class structures. Whiskers represent the 95% credible intervals. (*B*) The breakdown of *R*_S_ corresponding to each type of within-school relationships. Whiskers represent the 95% credible intervals. (*C*) Stacked graphs of *R*_S_ based on the median estimates.

The temporal clustering shown in Fig. 1*D* supports the hypothesis that the transmission is more likely within class, followed by within grade and within school. We explored this further by estimating reproduction numbers within school. Using a mathematical model that accounts for different levels of interaction within and between classrooms and grades as well as introductions from households and community, we estimated the within-school effective reproduction number *R*_S_ of seasonal influenza in primary schools along with the breakdown of transmission risks associated with class/grade relationships (Fig. 2). The relationship between any pair of students in the same school was classified as either “classmates,” “grademates” (in the same grade but not classmates), or “schoolmates” (not in the same grade). The estimated *R*_S_ was broken down as a sum of the contributions from these students, where the class size (*n*) and the number of classes per grade (*m*) were assumed to affect the risk of transmission. The reconstructed overall *R*_S_ in a 6-y primary school was estimated to be around 0.7 to 0.9 and was not significantly associated with *n* or *m* (Fig. 2*A*). Namely, an infected student was suggested to generate a similar number of secondary cases irrespective of the class structure; although our estimates of *R*_S_ were about 15% smaller for the class size of 40 than 20,* the posterior *P* value did not suggest a statistical significance (*p* ∼0.15 or above). As *R*_S_ was likely below 1 across class structures, school outbreaks may not have been sustained without continuous introductions from households and community. Transmission to classmates accounted for about two-thirds of *R*_S_ when each grade has only one class and was partially replaced by transmission to grademates as the number of classes per grade increases, while the sum of within-grade transmission (i.e., transmission to either classmates or grademates) remained stable (Figs. 2 *B* and *C*). Around 20 to 30% of overall *R*_S_ was explained by transmission to schoolmates throughout. We also obtained qualitatively similar results throughout our sensitivity analysis (*SI Appendix*, Fig. S4). In a 6-y school with three classes of 30 students, the risk of transmission was estimated to be 1.8% (95% credible interval [CrI]: 1.3 to 2.4) from a given infected classmate of the same sex, 1.6% (1.2 to 2.1) the opposite sex, 0.12% (0.08 to 0.19) from a given infected grademate, and 0.036% (0.026 to 0.049) from a given infected schoolmate (*SI Appendix*, Table S3). The cumulative risk of infection from the community was estimated to be 2.0% (1.6 to 2.5) over the season.

We incorporated a log-linear regression (35) into this estimation of *R*_S_ to account for covariates that may affect the susceptibility or infectiousness of students. The results suggested that vaccines were associated with reduced susceptibility, while mask-wearing was associated with both reduced susceptibility and infectiousness (Table 1). Conversely, hand washing was associated with increased susceptibility. Reduced chance of transmission during the winter break (27 December 2014 to 7 January 2015) was captured as a 76% estimated decline in the infectiousness of cases whose onset dates were during the break. School grade, which serves as a proxy of students’ age, did not show a significant association with either susceptibility (relative value 1.03; CrI: 0.98 to 1.09) or infectiousness (relative value 0.94; CrI: 0.88 to 1.00).

**Table 1.** Covariates and effects estimated in the log-linear regression

Covariate	Frequency in data	Relative susceptibility	Relative infectiousness
School grade (1 y increase)		1.03 (0.98 to 1.09)	0.94 (0.88 to 1.00)
Vaccine	47.7%	0.89* (0.81 to 0.97)	0.97 (0.81 to 1.14)
Mask-wearing	51.4%	0.77* (0.70 to 0.84)	0.66* (0.56 to 0.79)
Hand washing	80.1%	1.54* (1.36 to 1.75)	1.27 (0.97 to 1.72)
Onset in winter break	5.9% (of cases)		0.24* (0.14 to 0.37)

Values are median estimates and 95% credible intervals.

^*^Estimates with 95% credible intervals not crossing 1.

**Fig. 3.**
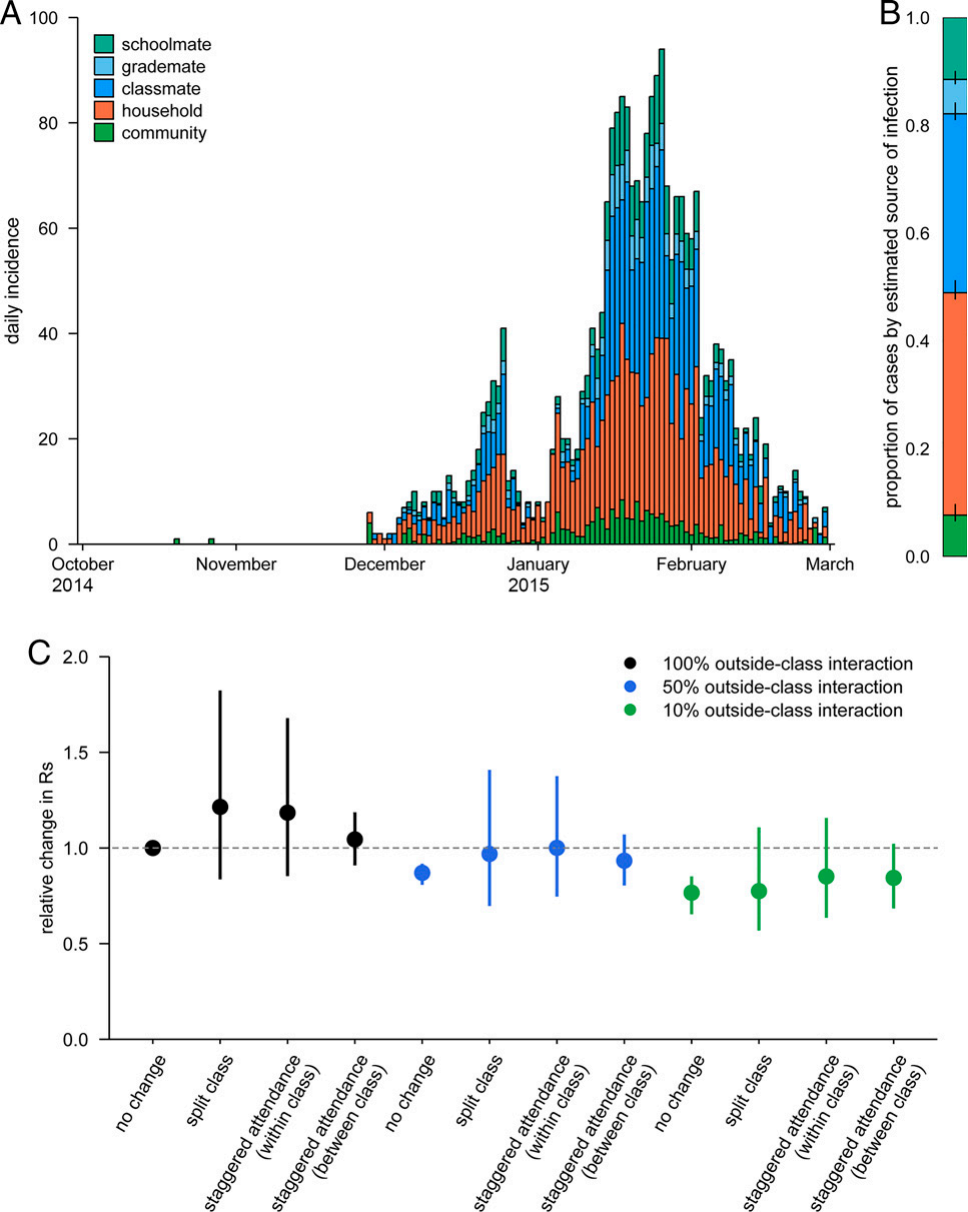
Reconstruction of students’ source of infection. (*A*) Epidemic curve stratified by the reconstructed source of infection. The conditional probability of infection from different sources was computed for each student and aggregated by date of illness onset. (*B*) Breakdown of the reconstructed source of infection. For each student, the source of infection was sampled based on the conditional probability to provide the proportion of students infected from each source. Bars denote posterior median and whiskers 95% credible intervals. (*C*) Expected relative changes in the school reproduction number under school-based interventions changing the structure of classes. Dots represent medians and whiskers 95% credible intervals. Reduced outside-class transmissions (i.e., from grademates or schoolmates) were also considered (50% reduction: blue; 90% reduction: green).

We estimated the breakdown of the source of infection for student cases based on the conditional probability predicted by the model and parameter estimates. The epidemic curve stratified by the estimated source of infection suggested that within-school transmission accounted for the majority of student cases while schools were open and that the within-household transmission was responsible for most of the cases reported during the winter break and shortly after (Fig. 3*A*). The aggregated relative contribution suggested that 51.1% (CrI: 50.0 to 52.0), 41.3% (CrI: 40.6 to 41.9), and 7.7% (CrI: 7.0 to 8.4) of the student cases were acquired from school, household, and community, respectively (Fig. 3*B*).

We estimated the possible relative effects of interventions altering the school population structure on the school reproduction number *R*_S_. We assumed that the estimated relative contributions of class/grade relationship to the transmission risk reflect the contact patterns between students which may also be relevant to the dynamics of another influenza outbreak at school (and potentially those of directly transmitted disease outbreaks in general) and that the responses to interventions can be captured by the estimated relationship between *R*_S_ and the changes in the variables *n* and *m* according to each intervention (Table 2). Specifically, in the “split class” scenario, each class was assumed to be split in half and taught simultaneously in separate classrooms, while in the “staggered attendance” scenarios, only half of the students attend school at the same time by introducing two different time schedules, e.g., morning and evening classes. The estimated relative effects of school-based interventions on *R*_S_ in a hypothetical setting of 6-y school with two classes per grade (40 students each) showed that splitting classes or staggered attendance alone was unlikely to reduce *R*_S_ (or may even be counteractive) (Fig. 1*D*), which is consistent with the aforementioned estimates of *R*_S_ minimally associated with class sizes and the number of classes. By reducing interactions between students from different classes (so-called “bubbling” or “cohorting”) by 90%, *R*_S_ could be reduced by up to around 20%. Combining split classes/staggered attendance with reduced interactions outside classes did not suggest incremental benefit in reducing *R*_S_. Given that these interventions typically require additional resources including staff and classrooms, the overall benefit to changing class structures for influenza control may be limited.

**Table 2.** Summary of interventions that changes the size/number of classes

Interventions	Class size (*n*)	No. classes per grade (*m*)	Assumption
Baseline (“no change”)	40	2	Students’ contacts within and between classes and grades are proportional to the estimated transmission patterns in Fig. 2.
Split class	20	4	Each class is split into two and taught simultaneously in separate classrooms. Students may contact each other between classes.
Staggered attendance (within class)	20	2	Each class is split into two and taught separately in two different time slots (e.g., morning and evening). Students in different time slots do not contact each other, and thus *R*_S_ is calculated for students in one slot.
Staggered attendance (between class)	40	1	Each class is allocated (as a whole) to either of the two different time slots and taught separately. Students in different time slots do not contact each other, and thus *R*_S_ is calculated for students in one slot.

## Discussion

We used a mathematical model that stratified transmission within and between classes/grades to understand the dynamics of influenza transmission among primary school students. The inferred transmission dynamics of seasonal influenza in Matsumoto city, Japan, in the 2014 to 2015 season suggested that the within-school reproduction number *R*_S_ stayed relatively constant regardless of the size or the number of classes [suggesting “frequency-dependent mixing” (17)], in contrast to common modeling assumptions. The estimated *R*_S_ of 0.8 to 0.9, more than half of which was attributable to within-class transmissions, is consistent with a previous study in the United States (15). This value is also in line with the reported *R*_0_ of 1.2 to 1.3 for seasonal influenza (36) because our previous study estimated that the students in this dataset had infected 0.3 to 0.4 household members on average during this 2014 to 2015 season (note that *R*_0_ corresponds to the overall number of secondary transmissions per student, including at school and household) (18). The value of *R*_S_ below 1 suggests that an outbreak cannot sustain itself within a school alone and that interactions through importing and exporting infections between households and the general community is likely to play a crucial role in the overall transmission dynamics. We estimated that school, household, and community accounted for 51%, 41%, and 8% of the source of infection for student cases, respectively. The attributable proportion was lower for schools and higher for households than the previous study (15), which may be explained by different scales of outbreaks in schools and households. In the Matsumoto city dataset, the overall attack ratio at school was lower (19%), students had larger households (average size 5.5), and there were more household cases than student cases (3,996 vs. 2,548), as opposed to 35%, size of 3.4 and 141 vs. 129 cases in ref. 15.

The estimated breakdown of *R*_S_ revealed a number of notable patterns. As the number of classes per grade increased, the contribution of within-class transmission risk declined and was replaced by within-grade transmission. Combined with the almost constant overall *R*_S_, this might indicate that contact behavior between students that contributed to transmission was only minimally affected by the student population density. That is, students may have had a certain number of “close friends” with whom they had more intimate interactions that could facilitate transmission. In a school with more classes per grade, some of such friendship may have come from grademates instead of classmates, but the total number of close friends may have remained similar. This interpretation is in line with published evidence of influenza spreading predominantly in close proximity (37) and is likely to influence the expected effect of interventions not only for influenza but also other respiratory infectious diseases including COVID-19, which share similar routes and range of transmission (38, 39). Further disease-specific studies could elucidate the generalizability of these associations in more detail.

Our results suggested that interventions such as reducing class sizes or the number of students present (staggered attendance) may not be effective in contrast to what would be expected under the density-dependent mixing assumption (27–31). If interventions altering class structures are not accompanied by additional precaution measures and students try to resume their “natural” behaviors (i.e., the same contact patterns as those in school with the resulting class structures) through so-called social contact “rewiring” (40), the effect of such interventions can diminish or even reverse. For example, if other classes are absent due to staggered attendance, students may increase their interactions with classmates instead of their previous close friends in other classes. Our results are also consistent with a recent study of interventions against COVID-19 in US schools that did not find a significant risk reduction associated with reducing class sizes (41). Given the additional logistical resources required to implement these interventions, we propose that reducing the class sizes or the number of attending students should be considered only if they enable effective implementation of precaution measures such as physical distancing, environmental cleaning, or forming social bubbles.

Using a log-linear regression analysis combined with a transmission model, we identified several precautionary measures associated with the susceptibility or infectiousness of students. Vaccines were associated with reduced susceptibility and masks with a reduction in both susceptibility and infectiousness. Influenza vaccine effectiveness in the 2014 to 2015 season was suggested to be particularly low in Japan due to vaccine mismatch and estimated to be 26% (95% CrI: 7 to 41%) for primary-school-age children (6 to 12 y old) (42). Our estimate of a relative susceptibility of 0.89 (CrI: 0.81 to 0.97) in vaccinated students, which translates into a vaccine effectiveness of 11% (CrI: 3 to 19%), is broadly consistent with this prior estimate. While existing evidence for the effectiveness of mask policies for the control of respiratory infections is still limited (43, 44), our estimates of small protective effects acting on the relative susceptibility (0.77; CrI: 0.70 to 0.84) and infectiousness (0.66; CrI: 0.56 to 0.79) lie within a plausible range based on evidence available to date (43, 45–47). Increased susceptibility associated with hand washing in our analysis, however, does not align with existing findings (48, 49). The amount of exposure (i.e., cases around a student) was explicitly adjusted for in our transmission model, limiting the possibility of typical confounding where hand-washing behavior was triggered by outbreak intensity. Although the underlying cause for this association is unclear, the original report on the Matsumoto city dataset also reported a higher odds ratio (1.4; CrI: 1.27 to 1.64; unadjusted for differential exposure) and attributed it to the possible congregation of students washing hands in communal settings at school (50).

Several limitations of this study should be noted. First, the transmission patterns within schools were estimated from a single dataset of seasonal influenza in primary schools (aged 5 to 12 y) in Matsumoto city, Japan, and it is unclear to what extent the results can be extrapolated to other settings, e.g., secondary schools or schools in other countries. Some features of our results may still be relevant to transmission dynamics in different types of schools if they reflect general social contact behaviors of schoolchildren; however, the relative contribution of within-class/within-grade interactions may become smaller for older students (13). The data points used in the inference mostly consisted of classes of size 20 to 40 (those with a size smaller than 10 were excluded, as they might be operated differently), and most schools had no more than 5 classes per grade. The scope of the estimated effect of the school-based interventions was also limited to within this range for internal consistency and thus may not necessarily be applicable to class structures outside this range (e.g., splitting a class of 20 students into two). Extrapolating the estimated transmission patterns to other respiratory infectious diseases also warrants caution because their epidemiological characteristics may not be identical, although we believe that such an approach may still be useful for diseases sharing similar modes of transmission. Modeling studies using social contact data often assume proportionality between contacts and the transmission of directly transmitted diseases (e.g., measles, influenza, and COVID-19) and have many successful applications (7, 33, 51–55). Using the estimated transmission patterns of influenza as a proxy for other diseases essentially rests on the same assumption, which nonetheless has limitations and should eventually be validated by disease-specific studies. Second, some aspect of the outbreaks may have been missing from the dataset. Since the illness data of teachers were not available, they were not considered throughout the analysis. However, their role in seasonal influenza transmission may have been minor given a large number of student cases and the smaller risk in adults (56, 57). Although our student incidence data likely had good case ascertainment given encouraged medical attendance and confirmation by rapid diagnostic kits (18), a certain proportion of infections (e.g., asymptomatic or very mild) may have been missing. We believe that students feeling unwell due to influenza mostly attended medical institutions and received a test as it was encouraged by schools. Nonetheless, it should be noted that this could have been a source of bias in the estimated transmission patterns. Students with very mild symptoms (e.g., only slightly sore throat) may visit a medical institution only if they know of other classmates also diagnosed with influenza. If such cases were common, the contribution of within-class transmissions in our results might have been an overestimate. Third, since the dataset was obtained from an observational study, the identified determinants of transmission may not be causal and should not be viewed as conclusive evidence. The results of our log-linear regression were mostly in line with existing findings; however, our dataset may still be biased due to unmeasured confounders such as health awareness. Our estimates of the relative effect of school-based interventions were based on the assumption that students’ behaviors follow the fixed patterns according to the school structure even under interventions. That is, when the class size or the number of classes were changed by an intervention, students were assumed to change their behavior according to the new school structure (as if it were the original structure) by, for example, rewiring close contacts in a timely manner. This is a hypothetical expectation that may not exactly be observed in actual interventional settings; for example, it may take time for students to resume close contacts after the class is split, which can bring *R*_S_ lower than our prediction at least temporarily. We have also neglected the possible effect of the interventions on the transmission outside the school. The actual effects of these interventions should ideally be validated by empirical data, as in ref. 41.

Our analysis disentangled the transmission dynamics of seasonal influenza among primary school students and highlighted the relative importance of within-class and within-grade transmission. Since class and school sizes were minimally associated with the within-school reproduction number, school-based interventions that change classroom structures, e.g., reduced class sizes and staggered attendance, may have limited effectiveness. Empirical evidence on fine-grained heterogeneous transmission patterns at school as was obtained from this study would inform public health planning for future outbreaks of influenza and, potentially, other directly transmitted infectious diseases that thrive in schools.

## Materials and Methods

### Data.

We analyzed a citywide school-based influenza survey data from the 2014/15 season. The survey was conducted in Matsumoto city [population size: 242,000 (58)], Japan, enrolling 13,217 students from all 29 public primary schools in the city. During the survey period (from October 2014 to February 2015), the participants were asked to fill out a questionnaire when they were back from the suspension of attendance due to diagnosed influenza (prospective survey). In March, the participants were asked to respond to another survey on their experience during the study period, regardless of whether they had contracted influenza (retrospective survey). A total of 2,548 diagnosed influenza episodes were reported in the prospective survey, which accounted for 96% of the cases officially recognized by the schools during the study period. Primary schools in Japan often requested students suspected of influenza to seek diagnosis at a medical institution. All students reporting an influenza episode in the prospective survey answered that they had received a diagnosis and at least 95% of them were noticed of type A influenza (indicating that they were laboratory-confirmed). In the retrospective survey, 11,390 (86%) participants responded, among which 8,375 reported that they did not have influenza during the study period.

We combined those who responded to the prospective survey (“case group”) and those who reported no influenza experience in the retrospective survey (“control group”) and obtained a dataset of 10,923 students. Of those, 71 students from 3 schools with less than 15 students per grade were excluded because they may have different schooling patterns from other schools (e.g., some students in different grades shared classrooms). We used individual profiles (sex, school, grade, class, household composition), onset dates, influenza episodes of household members, and precaution measures students engaged in (vaccine, mask, hand washing) in the subsequent analysis. Further details of the dataset can be found in the original studies (50, 59).

The secondary data analysis conducted in the present study was approved by the ethics committee at the London School of Hygiene and Tropical Medicine (reference number: 14599).

### Inference Model.

We modeled within-school transmission considering class structures as follows. We defined the “school proximity” *d* between a pair of students *i* and *j* attending the same school as

[1] $$d=\{\begin{array}{cc} 1 & \left(\text{different}\;\text{grades},\;\text{same}\;\text{school}\right)\\ 2 & \left(\text{different}\;\text{classes},\text{same}\;\text{grade}\right)\\ 3 & \left(\text{different}\;\text{sex},\;\text{same}\;\text{class}\right)\\ 4 & \left(\text{same}\;\text{sex},\;\text{same}\;\text{class}\right) \end{array}.$$

To investigate the potential effect of reduced class sizes and the number of attending students, we modeled the transmission between students as a function of two variables: the class size *n* and the number of classes per grade *m* (i.e., the number of students per grade is *nm*). Namely, we assumed that in the absence of any individual covariate effects, the cumulative transmission rate between student *i* and *j* in proximity *d* over the infectious period is represented as

[2] $$\beta_{ij}=\beta_{d}{\left(n_{i,d}\right)}^{-\gamma_{d}}{\left(m_{i,d}\right)}^{-\delta_{d}},$$

where $\beta_{d}$, $\gamma_{d}$, and $\delta_{d}$ are parameters to be estimated. When *i* and *j* are in the same grade (i.e., *d* = 2, 3, 4), the average class size and the number of classes in that grade were used as $n_{i,d}$ and $m_{i,d}$. When *d* = 1, the school average was used as $n_{i,d}$ and $m_{i,d}$. The exponent parameters within the same class were assumed to be equal: $\gamma_{3}=\gamma_{4}$ and $\delta_{3}=\delta_{4}$.

We modeled the daily hazard of incidence for student *i* as a renewal process. Let $h_{\tau }$ be the onset-based transmission hazard as a function of serial interval *s* (normalized such that $\overset{\infty }{\underset{s=1}{\sum }}h_{s}=1$; $h_{s}=0$ for *s* ≤ 0). We used a gamma distribution of a mean of 1.7 and an SD of 1.0 for influenza, which resulted in a mean serial interval of 2.2 d (60). The daily hazard of disease onset attributed to school transmission is given as

[3] $$\lambda_{i}^{\text{S}}\left(T\right)=v_{i}\underset{j}{\sum }w_{j}\beta_{ij}h_{T-T_{j}},$$

where *v_i_* and *w_i_* represent the relative susceptibility and infectiousness, respectively, which are specified for each individual by a log-linear regression model to account for covariates (see *SI Appendix* for detailed methods).

In addition to the within-school transmission, we also considered within-household transmission and general community transmission. Within-household transmission was incorporated as the Longini-Koopman model (61) using parameters from a previous study on the same cohort of students (18). General community transmission was modeled as a logistic curve fitted to the total incidence in the dataset to reflect the overall trend of the epidemic. See *SI Appendix* for further details of the model.

We constructed the likelihood function and estimated the parameters by the Markov-chain Monte Carlo (adaptive mixture Metropolis) method. We obtained 1,000 thinned samples from 250,000 iterations after 250,000 iterations of burn-in, which yielded the effective sample size of at least 300 for each parameter. Using the posterior samples, we computed the proximity-specific reproduction number *R_d_* in a hypothetical 6-y school with given *n* and *m* (assumed to be constant schoolwide) as

[4] $$R_{d}=\{\begin{array}{c} 5nm\cdot \beta_{1}n^{-\gamma_{1}}m^{-\delta_{1}}\;(d=1)\\ n(m-1)\cdot \beta_{2}n^{-\gamma_{2}}m^{-\delta_{2}}\;(d=2)\\ n\cdot \frac{\beta_{3}+\beta_{4}}{2}n^{-\gamma_{3}}m^{-\delta_{3}}\;(d=3,\;4) \end{array}$$

and defined the within-school reproduction number *R*_S_ as a sum of them.

We predicted the relative reduction in *R*_S_ under intervention measures changing the number of attending students and class structures by using posterior samples. Interventions were assumed to change *n* and *m* as shown in Table 1, and the predictive distribution of the relative change in *R*_S_ was computed for each intervention. The estimated *R*_S_ represents the value in a hypothetical condition in which an infectious student spends the whole infectious period at school; the effect of absence due to symptoms or the staggered attendance was not included in this reduction.

Further details of the model can be found in the *SI Appendix*, Text. All analysis was performed in Julia 1.5.2 and R 4.1.0. Replication code is available on GitHub (https://github.com/akira-endo/schooldynamics_FluMatsumoto14-15).

## Data Availability

Due to potentially sensitive information included, the original dataset is not made public and is available from the corresponding author upon reasonable request. A processed dataset with an increased level of anonymity, which can still qualitatively reproduce the main study finding (i.e., breakdown of the school reproduction number breakdown by the class/grade relationship without adjustment for covariates), is publicly available along with the accompanying code on a GitHub repository (https://github.com/akira-endo/schooldynamics_FluMatsumoto14-15), whose archived version at time of publication is available from ref. 62.

## Footnotes

^*^For example, the estimated relative reduction was 16% (95% credible interval: −18 to 42%) for m = 3.
